# Evaluation of NAFLD fibrosis, FIB-4 and APRI score in diabetic patients receiving exenatide treatment for non-alcoholic fatty liver disease

**DOI:** 10.1038/s41598-021-04361-x

**Published:** 2022-01-07

**Authors:** İlknur Ozturk Unsal, Murat Calapkulu, Muhammed Erkam Sencar, Basak Cakal, Mustafa Ozbek

**Affiliations:** 1 Department of Endocrinology and Metabolism, University of Health Sciences, Diskapi Yildirim Beyazit Training and Research Hospital, Sehit Omer Halisdemir Avenue, 06110 Ankara, Turkey; 2 Department of Gastroenterology, University of Health Sciences, Ankara Training and Research Hospital, Ankara, Turkey

**Keywords:** Endocrinology, Gastroenterology

## Abstract

There is a closely relationship between the development and progression of nonalcoholic fatty liver disease (NAFLD) or metabolic associated fatty liver disease (MAFLD) and obesity and diabetes. NAFLD fibrosis scores should be routinely used to rule out patients with advanced fibrosis. High scores may help identify patients at higher risk of all causes andliverrelated morbidity and mortality. The aim of this study was to investigate the association between exenatide and fibrosis scores. The effect of exenatide treatment on fibrosis scores was evaluated in type 2 diabetes mellitus (DM) patients with MAFLD. Evaluation was made of 50 patients with type 2 DM and MAFLD. The NFS, FIB4 and APRI scores were calculated before and after 6 months of treatment. After 6 months of exenatide treatment, the NFS and APRI scores were determined to have decreased significantly. Exenatide was observed to control blood glucose, reduce body weight and improve fibrosis scores in MAFLD patients with type 2 diabetes.

## Introduction

Non-alcoholic fatty liver disease (NAFLD) is the term used to encompass a range of conditions from simple steatosis to non-alcoholic steatohepatitis (NASH), which may progress to fibrosis and lead to liver cirrhosis in some patients^1,2^. The diagnosis of NAFLD is usually obtained from ultrasonography^3^. In the general population the prevalence of NAFLD has been reported to range from 20 to 40%, and it is estimated that NASH is seen at the rate of approximately 2–3%^2,4–6^. In 9–20% of NASH patients, there is progression to cirrhosis or hepatocellular carcinoma (HCC), and one-third die with various complications or will require liver transplantation^7,8^.

Recently, a consensus of international experts has proposed the disease name being changed from NAFLD to metabolic-associated fatty liver disease (MAFLD)^9–13^.

The development and progression of NAFLD is closely related to obesity and diabetes. Age, male gender, obesity, insulin resistance and the full spectrum of metabolic syndrome have been reported to be the leading risk factors for NAFLD^14^. The prevalence of NAFLD is 30–50% in patients with diabetes, and 80–90% in obese adults. With the global increase in type 2 DM and obesity, there is a currently increasing parallel prevalence of NAFLD^2^.

Type 2 DM is a chronic metabolic disease affecting more than 220 million individuals throughout the world^15^. The hormone, GLP-1, is known to reduce appetite and food intake together with the functions of stimulating insulin secretion, inhibiting glucagon secretion, and preventing gastric emptying and beta cell apoptosis^16^. Endogenous GLP-1 is broken down into inactive metabolites within 2–3 min by the enzyme dipeptidyl peptidase-4 (DPP-4). GLP-1 receptor agonists (GLP-1 RA) are resistant to this destruction. Exendin-4, whichhas been isolated from the saliva of the gila monster lizard, shows 53% sequence similarity to the initial 30 amino acids of human GLP-1 Synthetic exendin-4 (exenatide) is resistant to DPP-4 destruction^17,18^. Exenatide, which has a positive effect on glycemic control and provides weight loss, is used for diabetes treatment in overweight and obese adults with type 2 diabetes^19–21^. Furthermore, exenatide therapy has been shown to increase c-peptide levels, beta cell functions, and adiponectin levels^22^.

Recent studies have shown that exenatide reduces aminotransferase levels, and has a therapeutic effect on NAFLD in obese or overweight patients with type 2 DM^19,23–25^. However, the effects of exenatide treatment on fibrosis score remain unknown. The aim of the current study was to investigate the effect of 6 months of exenatide treatment on fibrosis scores in type 2 diabetic obese patients with NAFLD disease.

## Materials and methods

### Patient selection

**Figure 1:**
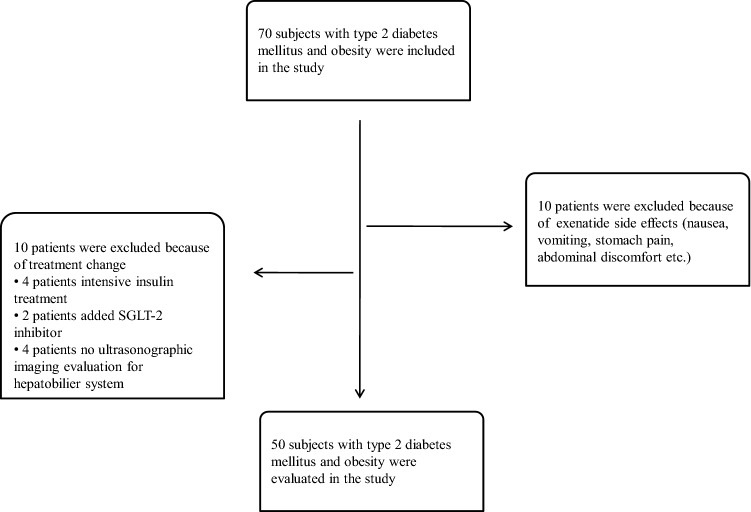
Study flow diagram.

This retrospective study included 70 patients who were diagnosed with type 2 DM and treated with exenatide plus metformin at our center between January 2017 and October 2019. All patients included in the study had body mass index (BMI) > 35 kg/m^2^, and were receiving metformin treatment agents because of the national healthcare system reimbursement conditions for exenatide in Turkey. NAFLD diagnosis was made from ultrasonographic findings. A total of 20 patients were excluded from the study; 10 with a change of treatment in the last 6 months and no ultrasonographic imaging before exenatide treatment, and 10 with drug intolerance due to side-effects (Fig. 1). All the subjects included in the study were prescribed a standard 1400 kcal diabetic diet in addition to 150 min/week mild-moderate exercise, and follow-up examinations were made at 3-month intervals. The initial exenatide treatment was at a dose of 5 μg twice a day, and this was increased to 10 μg twice daily after 4 weeks.

### Clinical and biochemical measurements

A record was made for all the study subjects of baseline demographic data, medications and clinical characteristics. Baseline and final follow-up values of weight, height, and BMI were recorded. The biochemical examination applied to all patients before and after the treatment included glycated haemoglobin (HbA1c), fasting plasma glucose (FPG), post-prandial glucose (PPG), creatinine, lipid profile, aspartate aminotransferase (AST), alanine aminotransferase (ALT), gamma-glutamyltransferase (GGT),and alkaline phosphatase (ALP). Blood samples were taken between 8:00 and 11:00 a.m. from each patient after a 10 h fasting. HbA1c was measured using the high-performance liquid chromatography (HPLC) method. Reference ranges were defined as FPG:74–100 mg/dl, AST: 0–40 U/l, ALT: 0–41 U/l, ALP: 40–129 U/l, GGT: 0–60 U/l, total cholesterol: 0–200 mg/dl, low-density lipoprotein cholesterol (LDL-C): 0–100 mg/dl, high-density lipoprotein cholesterol (HDL-C): 40–60 mg/dl, triglyceride: 0–200 mg/dl. The NAFLD score (NFS) was calculated based on age, body mass index, hyperglycemia or diabetes, AST/ALT, platelets, and albumin [= − 1.675 0.037 × age (years) 0.094 × BMI (kg/m^2^) 1.13 × IFG/diabetes (yes = 1, no = 0) 0.99 × AST/ALT ratio − 0.013 × platelet count (× 10^9^/l) − 0.66 × albumin (g/dl)]. The Fibrosis-4 index (FIB-4) index was calculated using the formula: Age (years) × AST (U/l)/[PLT(10^9^/l) × ALT^1/2^ (U/l). The AST to Platelet Ratio Index (APRI) is calculated using the formula: [(AST level/ULN)/platelet count (109/l)] × 100. The NFS scores were classified into three risk categories (low, intermediate, and high) according to cut-off points described in the original publications. These were < − 1.455 (F0–F2) and > 0.676 (F3–F4) for NFS^26–28^.

### Statistical analysis

Data obtained in the study were analysed statistically using SPSS version 23 software (IBM Corporation, NY, USA). Continuous variables are presented as mean ± standard deviation or median (min–max) values according to the distribution of the data. Categorical variables were presented as number (n) and percentage (%). The Dependent Sample t-test or the Wilcoxon test was used to compare numerical data in dependent groups, according to the conformity of the data to the normal distribution. The associations between categorical variables were performed using Chi-square analysis and the McNemar test. A value of p < 0.05 was accepted as statistically significant. The associations between numerical variables were analyzed using Pearson correlation analysis. Multivariate linear regression were carried out taking ΔNFS and ΔAPRI as the outcome variable and the changes in glycemic parameters and BMI as independent variables.

### Informed consent

This study conformed to the Helsinki Declaration. The study was approved by Dışkapı Yıldırım Beyazıt Training and Research Hospital Ethics Committee (No: 20.04.2020–86/13). All participants were informed about the research protocol, and they declared their voluntary attendance by signed written informed consent.

## Results

Evaluation was made of a total of 50 type 2 DM patients with NASH who had received exenatide treatment for six months. The patients comprised 39 (78%) females and 11 (22%) males with a mean age of 52.9 ± 9.8 years. The mean duration of diabetes was 10 ± 7.4 years. All the patients received metformin therapy, and 24 (48%) patients were using insulin therapy in addition to metformin. Medication for hypertension was being taken by 31 patients and their blood pressure was controlled with this medication. Abdominal ultrasonography (USG) was applied to all patients before starting exenatide treatment. The USG imaging revealed grade 1 hepatosteatosis (HS) in 5 (10%) patients, grade 2 HS in 34 (68%) patients, and grade 3 HS in 11 (%22) patients. The full demographic and baseline clinical data of the patients are reported in Table 1. There was a mean reduction of 8 kg in weight, and 3 kg/m^2^ in BMI in the 6th month. A significant decrease from the baseline values was determined for HbA1c (from 9.3 to 8%, p < 0.001), FPG (from 202 to 162 mg/dl, p < 0.001) and PPG (from 284 to 217 mg/dl, p = 0.001). After 6 months of exenatide treatment, the mean serum levels of triglyceride decreased significantly, but there was no significant change in HDL-C, LDL-C and total cholesterol levels. A significant decrease was determined in mean AST (from 25.6 to 21 U/l, p = 0.02) and ALT (from 29.4 to 21.7 U/l, p = 0.001) levels in the 6th month. GGT levels decreased significantly after exenatide treatment, and there was no significant change in the ALP level. NFS, FIB4 and APRI scores were calculated before and after 6 months of treatment. A significant decrease was determined in NFS (from − 0.007 to − 0.22, p = 0.002) and APRI score (from 0.23 to 0.20, p = 0.02) after 6 months of treatment and there was no significant change in FIB-4 score (Table 2). According to the NFS score, 10% (n:5) of patients were low-risk, 66% (n:33) were intermediate-risk, and 24% (n:12) were at high-risk before exenatide treatment. After six months of treatment, 26% (n:13) of patients were found to be low-risk, 60% (n:30) intermediate-risk, and 14% (n:7) high-risk. A significant decrease was found in the number of high and intermediate-risk patients compared to baseline values (from 90 to 74%, p = 0.008). As a result of the linear regression analysis, it was found that the effect of exenatide treatment on NFS (r2: 0.051, p = 0.208) and APRI (r2:0.028, p = 0.275) scores were independent of weight loss. When the patients evaluated according to BMI < 40 kg/m^2^ and > 40 kg/m^2^, significant difference in decrease HbA1c, and serum ALT level in both two groups found after 6 months of treatment. However, change in NFS score was found only group of BMI > 40 kg/m^2^ (Table 3). In the analysis after dividing the patients according to gender, there was a significant difference in serum ALT level, BMI, NFS and APRI scores in female after six months of treatment (Table 4). When patients were divided into 2 groups as HbA1c < 9% and > 9%, the difference was significant in terms of reduction in HbA1c, BMI, serum ALT level and NFS score in both groups after 6 months of treatment (Table 5). Multivariate linear regression analysis was performed for further analysis of whether weight loss or the changes in glycemic parameters were factors that might predict changes in fibrosis scores. There was no correlation between the in ΔNFS and ΔBMI (r:0.225, p:0.208) or ΔHbA1c (r:-0.108 p:0.535) values. No correlation was determined between the in ΔAPRI and ΔBMI (r:0.185, p:0.218) or ΔHbA1c (r:-0.004 p:0.978) values. Weight loss and the changes in glycemic parameters observed with exenatide treatment did not predict change in fibrosis scores (Table 6).

**Table 1:** Demographic and clinical data of the patients before and after exenatide therapy.

	Baseline	6. Month	p
Age (years)	52.9 ± 9.8		
Weight (kg)	109 ± 17	101 ± 15	< 0.001
BMI (kg/m^2^)	42.7 ± 5.8	40 ± 5.5	< 0.001
HbA1c (%)	9.3 ± 1.5	8 ± 1.7	< 0.001
FPG (mg/dl)	202 ± 64	162 ± 54	< 0.001
PPG (mg/dl)	284 ± 81	217 ± 69	0.001
AST (U/l)	25.6 ± 15.2	21 ± 7.3	0.017
ALT (U/l)	29.4 ± 16.5	21.7 ± 9.4	< 0.001
ALP (U/l)	90.3 ± 28.3	82.9 ± 29.1	0.08
GGT (U/l)	32 (17–211)	26 (13–144)	0.002
Creatinine (mg/dl)	0.86 ± 0.19	0.82 ± 0.17	0.06
T. cholesterol (mg/dl)	186.5 ± 44	181 ± 38.6	0.25
LDL-C (mg/dl)	127.6 ± 26.9	124.2 ± 32.9	0.26
HDL-C (mg/dl)	39.3 ± 8.1	41.1 ± 6.9	0.55
Triglyceride (mg/dl)	172 (63–1203)	149 (63–665)	0.02

*BMI* Body mass indeks, *FPG* Fasting plasma glucose, *PPG* Postprandial plasma glucose, *AST* Aspartate aminotransferase, *ALT* Alanine aminotransferase, *ALP* Alkaline phosphatase, *GGT* Gamma glutamyltransferase; *T. Cholesterol* Total cholesterol, *HDL-C* High-density lipoprotein cholesterol, *LDL-C* Low-density lipoprotein cholesterol.

**Table 2:** Evaluation of fibrosis score of the patients before and after exenatide therapy.

	Baseline	6. Month	p
NFS	− 0.07 (− 2.08–4.17)	− 0.22 (− 2.33–1.79)	0.002
FIB-4	0.77 (0.39–3.35)	0.82(0.39–2.34)	0.86
APRI	0.23 ± 0.15	0.20 ± 0.1	0.02

*NFS* NAFLD fibrosiz score, *FIB-4* Fibrosis-4 index, *APRI* AST to Platelet Ratio Index.

**Table 3:** Demographic and clinical data of the patients before and after exenatide therapy according to BMI.

Parameters	BMI < 40 kg/m^2^ (n:18)			BMI > 40 kg/m^2^(n:32)		
	Baseline	6. Month	p	Baseline	6. Month	p
HbA1c (%)	8.9 ± 1.4	8.1 ± 1.5	0.047	9.3 ± 1.6	7.9 ± 1.8	< 0.001
AST (U/l)	25.3 ± 14.2	19.9 ± 5.6	0.049	26.2 ± 16.3	22.1 ± 8.2	0.120
ALT (U/l)	30.1 ± 17	21.5 ± 8.1	0.004	29.5 ± 16.7	22 ± 10.1	0.005
ALP (U/l)	75.7 ± 27.6	78.2 ± 19.1	0.80	101.8 ± 33.9	87.6 ± 35.4	0.031
GGT (U/l)	30 (17–74)	27 (18–61)	0.399	34.5 (17–211)	27 (13–144)	0.005
Creatine (mg/dl)	0.89 ± 0.1	0.84 ± 0.1	0.008	0.83 ± 0.19	0.81 ± 0.18	0.46
T.C(mg/dl)	194.9 ± 40.7	188.9 ± 38.7	0.649	184.7 ± 35.3	176 ± 38.7	0.258
LDL (mg/dl)	133.4 ± 22.7	132.5 ± 38	0.878	124.5 ± 30.5	120.2 ± 29.7	0.243
HDL (mg/dl)	38.9 ± 8.1	40.5 ± 8.1	0.167	39.8 ± 8.1	39.9 ± 5.5	0.935
Triglyceride (mg/dl)	171 (98–1203)	148 (74–607)	0.255	177.5 (63–779)	162 (63–665)	0.063
NFS	− 1.08 (− 2.08–1.61)	− 0.22 (− 2.33–0.65)	0.214	− 0.045 (− 1.23–4.17)	− 0.19 (− 1.55–1.79)	0.004
APRI	0.25 ± 0.17	0.2 ± 0.08	0.071	0.22 ± 0.13	0.19 ± 0.11	0.217

*BMI* Body mass indeks, *AST* aspartate aminotransferase, *ALT* alanine aminotransferase, *ALP* alkaline phosphatase, *GGT* gamma glutamyltransferase; *T.C* total cholesterol, *HDL* High-density lipoprotein, *LDL* low-density lipoprotein cholesterol, *NFS* NAFLD fibrosizscore, *APRI* AST to Platelet Ratio Index.

**Table 4:** Demographic and clinical data of the patients before and after exenatide therapy according to gender.

Parameters	Male (n:11)			Female (n:39)		
	Baseline	6. Month	p	Baseline	6. Month	p
BMI (kg/m^2^)	39.7 (35.7–52)	39 (31.6–52)	0.183	41.9 (35.8–58)	39 (29.7–54)	< 0.001
HbA1c (%)	9.1 (6.9–11.7)	6.8 (5.2–12.4)	0.022	9.2 (6.7–13.1)	8.1 (5.6–13.1)	< 0.001
AST (U/l)	22 (15–64)	20 (14–36)	0.109	21.5 (11–98)	18.5 (11–39)	0.053
ALT (U/l)	29 (19–45)	24 (15–43)	0.023	25 (13–99)	18 (11–46)	< 0.001
ALP (U/l)	77.5 (50–94)	58 (56–69)	0.180	94.5 (19–167)	85 (17–155)	0.064
GGT (U/l)	31 (19–92)	27 (19–35)	0.414	32 (17–211)	25 (13–144)	0.002
Creatine (mg/dl)	0.94 (0.8–1.2)	0.86 (0.69–1.1)	0.013	0.78 (0.59–1.4)	0.77 (0.51–1.3)	0.150
T.C (mg/dl)	183 (125–333)	186 (155–217)	0.753	187 (109–270)	168 (116–256)	0.319
LDL (mg/dl)	127 (80–185)	140 (68–210)	0.859	131 (81–193)	115 (72–208)	0.121
HDL (mg/dl)	33 (30–43)	38 (30–54)	0.114	41 (24–56)	41 (30–55)	0.080
Triglyceride (mg/dl)	165 (102–1203)	165 (88–607)	0.257	174 (63–779)	148 (63–665)	0.951
NFS	0.85 (− 0.16–1.61)	0.385 (− 0.13–1.43)	0.173	− 0.09 (− 2.08–4.17)	− 0.34 (− 2.33–1.79)	0.009
APRI	0.1955 (0.12–0.77)	0.185 (0.12–0.38)	0.386	0.167 (0.07–0.7)	0.1695 (0.07–0.53)	0.036

*BMI* Body mass indeks, *AST* Aspartate aminotransferase, *ALT* Alanine aminotransferase, *ALP* alkaline phosphatase, *GGT* Gamma glutamyltransferase, *T.C* Total cholesterol, *HDL* High-density lipoprotein, *LDL* Low-density lipoprotein, *NFS* NAFLD fibrosiz score, *APRI* AST to Platelet Ratio Index.

**Table 5:** Demographic and clinical data of the patients before and after exenatide therapy into HbA1c.

Parameters	HbA1c < 9% (n:23)			HbA1c > 9% (n:27)		
	Baseline	6. Month	p	Baseline	6. Month	p
BMI (kg/m^2^)	41.5 ± 5.4	38.8 ± 4.8	< 0.001	43.5 ± 6.3	41.1 ± 5.9	0.001
AST (U/l)	31.3 ± 18.9	24.1 ± 7.2	0.037	19.6 ± 5.7	17.9 ± 6.3	0.209
ALT (U/l)	35.6 ± 20.5	26.3 ± 9.9	0.013	24.1 ± 9.4	17.6 ± 6.8	< 0.001
ALP (U/l)	92.5 ± 36	85.1 ± 36.2	0.408	92.6 ± 31.7	81.6 ± 25.1	0.056
GGT (U/l)	38 (17–211)	36 (24–144)	0.013	25 (17–92)	20 (13–47)	0.035
Creatine (mg/dl)	0.84 ± 0.21	0.81 ± 0.15	0.227	0.87 ± 0.16	0.83 ± 0.18	0.176
T.C (mg/dl)	180 ± 34.8	175 ± 38.5	0.508	197.2 ± 38.9	187.3 ± 38	0.373
LDL (mg/dl)	125.1 ± 28.5	124.7 ± 31.5	0.906	129.7 ± 27.7	123.8 ± 34.7	0.213
HDL (mg/dl)	39.6 ± 7.6	40.6 ± 7.2	0.544	39.3 ± 8.6	39.7 ± 5.8	0.0808
Triglyceride (mg/dl)	167 (79–779)	146 (63–665)	0.163	184 (63–1203)	151 (85–607)	0.071
NFS	− 0.09 (− 1.91–1.85)	− 0.38 (− 2.33–1.75)	0.047	− 0.045 (− 2.08–4.17)	− 0.19 (− 1.9–1.79)	0.012
APRI	0.29 ± 0.18	0.24 ± 0.11	0.168	0.18 ± 0.09	0.15 ± 0.06	0.072

*BMI* Body mass indeks, *AST* Aspartate aminotransferase, *ALT* Alanine aminotransferase, *ALP* Alkaline phosphatase, *GGT* Gamma glutamyltransferase, *T.C* Total cholesterol, *HDL* High-density lipoprotein, *LDL* Low-density lipoprotein, *NFS* NAFLD fibrosizscore, *APRI* AST to Platelet Ratio Index.

**Table 6:** The multivariate lineer regression analysis in all subjects of the study.

Variables	Predictors	β	Signficance	Lower bound of 95% CI for β	Upper bound of 95% CI for β
Δ NFS R^2^ = 0.138	Constant	0.134	0.354	− 0.165	0.433
	Δ HbA1c	0.065	0.396	− 0.093	0.223
	Δ BMI	0.019	0.689	− 0.078	0.116
	Δ FPG	− 0.001	0.716	− 0.008	0.006
	Δ PPG	− 0.002	0.313	− 0.006	0.002
Δ APRI R^2^ = 0.016	Constant	0.002	0.951	− 0.059	0.063
	Δ HbA1c	0.001	0.962	− 0.032	0.034
	Δ BMI	− 0.001	0.853	− 0.017	0.014
	Δ FPG	< 0.001	0.577	− 0.001	0.001
	Δ PPG	< 0.001	0.633	− 0.001	0.001

*Δ* Difference from baseline after exenatide treatment, *NFS* NAFLD fibrosiz score, *APRI* AST to Platelet Ratio Index, *BMI* Body mass indeks, *FPG* Fasting plasma glucose, *PPG* Postprandial plasma glucose.

## Discussion

NAFLD is a major cause of end-stage liver disease, HCC, and liver transplantation throughout the world. The pathogenesis of non-alcoholic fatty liver disease has not been exactly explained, but insulin resistance is known to have a key role in the development of fatty liver disease and potentially, steatohepatitis^29^^.^. Significantly elevated free fatty acids levels have been reported in NAFLD and type 2 diabetes mellitus cases, compared ro those with type 2 diabetes without NAFLD^30^. Exenatide has been shown to improve hepatic steatosis by increasing lipid use in adipose tissue in non-diabetic rats^31^. Although the mechanism is still not understood by which GLP-1 analogs improve hepatic steatosis, alterations in hepatic lipid metabolism have been thought to be a contributory factor to these effects^32^. Irrespective of insulin resistance, visceral fat has also been related to liver inflammation and fibrosis in NASH patients, and it is thought that this effect may be mediated by interleukin-6^33^. Hepatic lipid metabolism is affected by intrahepatic lipogenesis and lipolysis, and changes in the use of lipids in skeletal muscle and adipose tissue.

Conventional ultrasonography is low cost, safe, and accessible, and the most commonly used imaging technique for screening for fatty liver^34^. In the current study, USG imaging revealed grade 1 hepatosteatosis in 5 (10%) patients, grade 2 in 34 (68%) patients, and grade 3 in 11 (%22) patients. A meta-analysis showed that liver ultrasonography allows for reliable and accurate detection of moderate-severe fatty liver compared to histology with sensitivity and specificity of 84.8% and 93.6%, respectively^35^. Moreover, European guidelines recommend using ultrasonography as first-choice imaging for identify risk of NAFLD in adults^3^.

In the management of NAFLD, it is important to identify patients with a higher risk of NASH and advanced fibrosis. Liver biopsy is still required to identify fibrosis status and patients with NASH. However, liver biopsy is invasive, costly, less suitable for population-level screening, and shows inter-observer variability^28^. Noninvasive evaluation of fibrosis severity is important in management of NAFLD patients, because of the fibrosis stage is a determinant of mortality. Therefore, to identify NASH in patients with NAFLD, various clinical and biochemical models have been used.There has been shown to be an association between aminotransferases, and some histological parameters such as inflammation and steatosis^36^. Patients with NAFLD may have mild or moderate elevations in AST and ALT, although normal aminotransferase levels do not exclude NAFLD. Elevated AST and ALT values have been shown to usually be 2–5 times the upper limit of normal, giving an AST to ALT ratio of < 1, which differs from alcoholic fatty liver disease. However, the extent of liver transaminases elevation does not predict the extent of hepatic inflammation or fibrosis, and an ALTvalue within normal limits does not exclude histological injury of clinical importance. Thus, aminotransferase levels do not correlate with the degree of fibrosis^37–40^. In the current study patients, the initial AST and ALT values were within normal limits (25.6 U/l, 29.4 U/l, respectively). However, there was a significant decrease in mean AST and ALT levels in the 6th month of exenatide treatment. Buse et al. demonstrated that 2 years of exenatide therapy was associated with significant improvement in aminotransferase levels^41^. In previous studies, new normal ALT cut-off values (30 U/l for males and19 U/l for females) have been found to be associated with NASH. In the current study, 78% of the patients were female. In this case, the initial ALT level is at an acceptable level above normal. ALP may be elevated to two to three times the upper limit of normal. In the current study, ALP was normal and there was no significant change in the ALP level after exenatide treatment. GGT levels were observed to decrease significantly after exenatide treatment. Studies have shown that the serum GGT level in particular is a marker of oxidative stress rather than a specific marker of NAFLD-related liver disease. Although the key mechanism leading to NAFLD and perhaps NASH is insulin resistance, additional oxidative injury is required for the necrosis and inflammatory process in steatohepatitis^42^. In a retrospective case-series study, once weekly dulaglutide (0.75 mg) administration for 12 weeks was able to reduced serum transaminases levels and liver stiffness. 29 weeks treatment with lixisenatide increased the proportion of patients with normalisation of ALT in overweight/obese NAFLD subjects. A small randomized clinical trials showed that, liraglutide (3 mg daily) administration for 26 weeks reduced serum ALT and AST on obese NAFLD subjects without T2DM. According to studies using different formulations of GLP-1 analogues on NAFLD and NAHS obtained similar efficacy, a class effect on improving liver function^43^. After the 12-week treatment, ALT level (< 40 U/l) was in the exenatide group was significantly lower than in the metformin group in a study^25^. In the other study, levels of the hepatic injury biomarkers ALT, AST and GGT were significantly decreased in the exenatide group versus ınsulin group after 12 weeks. Exenatide can reduce apoptosis, oxidative stress and endoplasmic reticulum stress response and act directly on the liver, protecting it from various stress responses^24^.

GLP-1 RA has significant effects on glycemic control and weight loss. LEAN trial demonstrated that patients who used liraglutide underwent end-of-treatment liver biopsy had histological resolution in non-alcoholic steatohepatitis compared with placebo group^44^. In the phase 2 study, semaglutide at a daily dose of 0.4 mg significantly induced resolution of NASH without worsening of fibrosis after 72 weeks of treatment, but it did not induce an improvement of fibrosis stage > 1^45,46^.

For most patients with NAFLD, weight loss is the primary therapy. In a previous study, it was reported that steatosis was improved in 10 (20.4%) of 49 NAFLD patients with type 2 DM through treatment with exenatide plus modifications to lifestyle, although the rate of improvement showed no difference from that observed with treatment of metformin plus lifestyle modification^25^. In that study, BMI was < 30 kg/m^2^. In the current study, although BMI was > 40 kg/m^2^ after treatment, a decrease was observed in fibrosis scores with the use of exenatide. In another study of 30 NAFLD patients with elevated liver enzymes, obesity and type 2 DM, exenatide plus insulin was seen to improve steatosis from baseline in 28 (93.3%) patients, which was a significantly higher improvement rate than that observed with intensive insulin therapy^24^. Therefore, although initial therapy for type 2 DM is typically with metformin, the presence of NAFLD can be decisive in the choice of glucose lowering therapy.

Non-invasive fibrosis scores help distinguish patients with NAFLD who are at increased risk of liver-related complications or death. There are now several non-invasive methods to detect fibrosis in patients with liver disease. One of the scores, the NFS, is specific to NAFLD. Studies have suggested that higher NFS may be associated with increased mortality from cardiovascular disease^47^. In some studies have been shown that the Fib-4 score have good predictive accuracy for advanced fibrosis in patients with chronic hepatitis C virus (HCV) infection^48^. In another study, it performed better than other serological markers for predicting advanced fibrosis in patients with NAFLD. The APRI has firstly been evaluated in patients with HCV and human immunodeficiency virus or alcoholic liver disease. The ability of the APRI to predict outcomes in patients with NAFLD was examined in a retrospective series with 320 patients and it was determined that only the high-risk group was at greater risk of death or liver transplantation^49^.

The results of this study showed that the NFS, and APRI score decreased significantly after 6 months of exenatide treatment, but there was no significant change in FIB-4 score. According to the NFS score, the number of high and intermediate-risk patients decreased significantly compared to the baseline values. These non-invasive scoring systems, including NFS, FIB-4 index, and APRI index yield high negative predictive values but poor positive predictive values. Although NFS and FIB-4 display good diagnostic efficacy, many factors such as age, diabetes, may influence their diagnostic performance. Previous studies have shown that high fibrosis scores are associated with an increased risk of cardiovascular and liver-related mortality. Therefore, these scoring systems are used to exclude subjects without advanced fibrosis, thereby prevented unnecessary liver biopsies^28^.

In this clinical trial, there was seen to be a mean reduction of 8 kg in weight, and 3 kg/m^2^ in BMI after 6 months of exenatide treatment. The GLP-1 analogue may lead to improvements in fatty liver and insulin resistance. Although exenatide may not directly an insulin sensitizer agent, it can resulting in an insulin-sensitizing effect by significant weight loss. One possible hypothesis to explain this is that GLP-1 may have a direct effect on hepatocytes and affect the expression of genes such as PPAR-α and PPAR-gene connected to β-oxidation and insulin sensitivity of fatty acids^25^. HbA1c, FPG, PPG, and the mean serum levels of triglyceride significantly decreased from the baseline values after 6 months. In a study which a non-diabetic, HFD-induced rat NAFLD model was used the researchers showed that exenatide reduced both lipid deposition in the liver and adipose tissue and decreased the size of adipocytes^31^. Tushuizen et al. examined the effect of exenatide treatment on hepatosteatosis with Proton Magnetic Resonance Spectroscopy, and reported that after 44 weeks of treatment was reduction in a liver fat from 16.0 to 5.4%^50^. In another case series of 8 patients with type 2 DM and biopsy-proven NAFLD, after 28 weeks of exenatide therapy, the liver histology improved in 3 patients^23^.

The main limitation of this study was the retrospective design, unequal number of patients by gender, and that the liver biopsy could not be evaluated. Although there are studies of exenatide and NAFLD in literature, this is the first study to have evaluated the relationship between exenatide treatment and fibrosis scores in diabetic and obese patients.

In conclusion, exenatide may not only reduce blood glucose and body weight, but also improve fibrosis scores, reduce aminotransferase, and extenuate NAFLD in diabetic patients. The improvement of liver histology is the gold standard in the evaluation of therapeutic efficacy in patients with NAFLD. Therefore, it should be supported by studies with larger sample sizes in patients with biopsy-proven NASH.
